# Transactions of the Missouri Medical Association of the State of Missouri, at Its Second Annual Meeting, June, 1852

**Published:** 1852-10

**Authors:** 


Art. V.—*Transactions of the Missouri Medical Association of the State of Missouri, at its Second Annaal Meeting*, St Louis, 1852.

This is the second volume of Transactions of this Society, and a very creditable one is it to the industry and attainments of its members. Besides the minutes of proceedings, it consists of nearly a hundred pages of excellent matter, the annual address of the President, reports of Committees, essays, &c., and presents an appearance altogether respectable and tasteful. It is grateful to see such a production emanating from a part of the country where, in the recollection of some of the members of the Society, there could hardly be said to be a medical profession. Missouri, in every way, is making rapid strides to greatness, and our brethren there seem resolved that their profession shall keep pace with the commonwealth in commerce, wealth, and arts. Such a Society as they have organized reflects credit upon the State. We hope to find time to return to its Transactions, and to notice at length the carefully prepared and instructive reports and essays; but at present we can only commend them in general terms.

Dr. McPheeters delivered the annual address, of which his subject was, "*A thorough organization of the profession the only feasible plan of bringing about medical reform.*" Its tone and spirit are admirable, and it is written in an agreeable and graceful style. The reports on Surgery and Obstetrics, by Drs. Pope and Pallen, are excellent, and the same is true of all the others in the volume.

